# Impact of a Collective Intelligence Tailored Messaging System on Smoking Cessation: The Perspect Randomized Experiment

**DOI:** 10.2196/jmir.6465

**Published:** 2016-11-08

**Authors:** Rajani Shankar Sadasivam, Erin M Borglund, Roy Adams, Benjamin M Marlin, Thomas K Houston

**Affiliations:** ^1^ Division of Health Informatics and Implementation Science Quantitative Health Sciences University of Massachusetts Medical Scool Worcester, MA United States; ^2^ College of Information and Computer Sciences University of Massaachusttes Amherst Amherst, MA United States; ^3^ Center for Healthcare Organization and Implementation Research US Department Veterans Affairs Bedford VA Medical Center Bedford, MA United States

**Keywords:** recommender system, health communication, computer tailoring, smoking cessation

## Abstract

**Background:**

Outside health care, content tailoring is driven algorithmically using machine learning compared to the rule-based approach used in current implementations of computer-tailored health communication (CTHC) systems. A special class of machine learning systems (“recommender systems”) are used to select messages by combining the collective intelligence of their users (ie, the observed and inferred preferences of users as they interact with the system) and their user profiles. However, this approach has not been adequately tested for CTHC.

**Objective:**

Our aim was to compare, in a randomized experiment, a standard, evidence-based, rule-based CTHC (standard CTHC) to a novel machine learning CTHC: Patient Experience Recommender System for Persuasive Communication Tailoring (PERSPeCT). We hypothesized that PERSPeCT will select messages of higher influence than our standard CTHC system. This standard CTHC was proven effective in motivating smoking cessation in a prior randomized trial of 900 smokers (OR 1.70, 95% CI 1.03-2.81).

**Methods:**

PERSPeCT is an innovative hybrid machine learning recommender system that selects and sends motivational messages using algorithms that learn from message ratings from 846 previous participants (explicit feedback), and the prior explicit ratings of each individual participant. Current smokers (N=120) aged 18 years or older, English speaking, with Internet access were eligible to participate. These smokers were randomized to receive either PERSPeCT (intervention, n=74) or standard CTHC tailored messages (n=46). The study was conducted between October 2014 and January 2015. By randomization, we compared daily message ratings (mean of smoker ratings each day). At 30 days, we assessed the intervention’s perceived influence, 30-day cessation, and changes in readiness to quit from baseline.

**Results:**

The proportion of days when smokers agreed/strongly agreed (daily rating ≥4) that the messages influenced them to quit was significantly higher for PERSPeCT (73%, 23/30) than standard CTHC (44%, 14/30, *P*=.02). Among less educated smokers (n=49), this difference was even more pronounced for days strongly agree (intervention: 77%, 23/30; comparison: 23%, 7/30, *P*<.001). There was no significant difference in the frequency which PERSPeCT randomized smokers agreed or strongly agreed that the intervention influenced them to quit smoking (*P*=.07) and use nicotine replacement therapy (*P*=.09). Among those who completed follow-up, 36% (20/55) of PERSPeCT smokers and 32% (11/34) of the standard CTHC group stopped smoking for one day or longer (*P*=.70).

**Conclusions:**

Compared to standard CTHC with proven effectiveness, PERSPeCT outperformed in terms of influence ratings and resulted in similar cessation rates.

**ClinicalTrial:**

Clinicaltrials.gov NCT02200432; https://clinicaltrials.gov/ct2/show/NCT02200432 (Archived by WebCite at http://www.webcitation.org/6lEJY1KEd)

## Introduction

In computer-tailored health communication (CTHC) systems, messages are tailored (what messages need to be selected for the patient) to patient characteristics [1]. Across health domains, CTHC systems are effective in motivating behavior change [2-8]. In the smoking cessation domain, meta-analyses have demonstrated the effectiveness of CTHC systems [9]. In a previous randomized controlled trial (RCT; N=900), we developed and demonstrated the effectiveness of a CTHC system. Compared with an active control group that received no messages, this CTHC system significantly impacted 6-month cessation outcomes (OR 1.70, 95% CI 1.03-2.81) [10]. Current implementations of CTHC systems (hereafter referred to as “standard CTHC”) combine tailoring variables (what variables should be used to tailor) and if-then-else rules (how to select messages for the different tailoring variables) to select messages for a patient [1,11]. Experts (or study designers) specify these tailoring variables and develop the rules based on their knowledge of the targeted population, literature, and health behavior theories.

Outside health care, content tailoring is driven algorithmically using machine learning as opposed to the rule-based approach used in standard CTHC systems [12-14]. A special class of machine learning systems (“recommender systems”) are used to select messages combining the collective intelligence of their users (ie, the observed and inferred preferences of users as they interact with the system) and their user profiles [12-14]. For example, Amazon recommends products that a customer may like based on the products they have purchased or viewed previously. The primary difference between standard CTHC and recommender systems is how the messages are selected. As noted, in standard CTHC systems, messages are selected using if-then-else rules. In recommender systems, machine learning algorithms select the messages. As published, recommender systems offer multiple potential advantages to CTHC including the ability to continually learn and adapt to user feedback; however, this approach has not been adequately tested for CTHC [11].

In an experiment funded by the Patient-Centered Outcomes Research Institute (PCORI), we developed and evaluated a recommender system, the Patient Experience Recommender System for Persuasive Communication Tailoring (PERSPeCT), and applied it to smoking cessation. We compare PERSPeCT with our existing, evidence-based, standard rule-based CTHC system that was demonstrated to be effective in our previous RCT [10]. Our primary hypothesis is that the PERSPeCT recommender system will outperform (ie, select messages of higher influence) the rule-based CTHC system. We also evaluate the perceived intervention influence and 30-day cessation at follow-up. Our study provides the first evidence for the use of machine learning recommender systems for motivating smokers, and has important implications for future behavioral interventions.

## Methods

### Study Overview

In a randomized experiment, we compared PERSPeCT (intervention) with a standard rule-based CTHC system (comparison). As noted previously, the purpose of this pilot experiment was to test whether selecting the messages by a recommender approach would provide marginal advances over a standard message selection approach. As noted, this comparison system tailored messages based on the smoker’s readiness to quit and was demonstrated to be effective for smoking cessation in our previous RCT [10]. For the PERSPeCT intervention, we developed and implemented a recommender system [15,16]. Both the comparison and intervention system drew from the same motivational message content, but varied in how messages were selected for each participant. Messages were sent until the smoker entered ratings for 30 messages. Smokers in both arms were emailed daily motivational messages and were incentivized to rate messages. The study was conducted between October 2014 and January 2015. Our protocol is described in detail subsequently. This study was approved by the University of Massachusetts Medical School Institutional Review Board. See Multimedia Appendix 1.

### The PERSPeCT Intervention and Comparison Standard System

Our study goal was to test the ability of the two systems to select influential messages for individual participants. Thus, for both the intervention and comparison systems we used the same message database. In this section, we first describe the messaging database used by both systems and then the PERSPeCT recommender and comparison rule-based standard CTHC system.

#### The Motivational Messaging Database

The messaging database included 261 messages that were developed in our previous RCT and included both expert-written messages and peer-written messages [17]. Messages written by experts (study designers, behaviorists, physicians, nurses) were developed through an iterative expert group review process. The creation of these messages was informed by current guidelines [18] and Social Cognitive Theory (SCT) [19]. The current guidelines provided evidence-based content on successful cessation strategies. The SCT, which incorporates vicarious learning and verbal persuasion, informed the content of the expert messages [17]. Messages reflected theoretical determinants of quitting, such as positive outcome expectations and self-efficacy enhancing small goals [19]. Peer-written messages were written by current and former smokers responding to an online survey that presented four scenarios tailored by gender, age, and readiness to quit, and solicited their responses. These messages were then reviewed for use in our system. More details of our methodology to generate peer-written messages have been previously published [17]. Peer-written messages included the more “social” and “real-life” aspects of smoking cessation and represented the day-to-day issues associated with smoking cessation and the social and interpersonal influences on quitting. Such messages align with the concepts of SCT in which the physical and social environment influences individual behavior change [17].

#### The Comparison: An Evidence-Based, Effective, Standard Computer-Tailored Health Communication System

As noted, our comparison standard CTHC was a rule-based system that tailored messages based on a smoker’s readiness to quit. We had previously demonstrated this system to be effective in a large, nationwide RCT (N=900) compared to a robust website control without tailored messages. This website control included such functions as risk, decisional balance, cessation barrier calculators, games linking the chemicals in smoking with their other uses (eg, formaldehyde is used in both cigarettes and in embalming), and a library of informational resources about smoking [10]. In the RCT, two emails were sent in the first 2 weeks, followed by one email every week until 6 months postregistration. Using a 6-month, 7-day point prevalence cessation outcome, smokers who received the motivational messages were assessed to be more likely to quit than those smokers who received the control website (OR 1.69, 95% CI 1.03-2.80) [10]. For this study, we again used this standard CTHC and messages were sent daily to smokers.

We selected this system as our comparison for multiple reasons. Firstly, it allowed isolating the effect of the message selection because the motivational messages’ content was the same for both systems. If we compared it to another system with different motivational messages content, estimating whether the differences between the two groups were due to message selection or the content of the two systems would have been challenging. Moreover, using an effective system provided a rigorous comparison for our system. At the time of the study design, there was no other online motivational messaging system with this level of effectiveness data.

#### The Intervention: The PERSPeCT Recommender System

The only difference between the comparison and intervention conditions was that the intervention smokers received motivational messages tailored by the PERSPeCT recommender system. Recommender systems can be implemented using either a content-based [20], collaborative filtering [21] or a hybrid approach [22]. PERSPeCT was implemented as a hybrid recommender system. Given a sample of rating data, content-based recommender systems can learn a function and match users to items based on the provided user profile information (ie, age, gender) and the metadata description of the item or message. Metadata is defined as data about data; it describes the structure or content of a particular resource, object, or entity [23]. Our coding of the messages by the readiness to quit categories in the comparison standard CTHC system is an example of the type of metadata that can be used by content-based recommender systems. Content-based recommender systems work similarly to standard CTHC systems, but the matching function can be optimized based on rating data instead of specified by experts.

In contrast to content-based recommender systems, collaborative filtering recommender systems match users to items based on past rating history. The simplest examples of this approach are nearest-neighbor methods [21]. These methods match a target user with other users that have given similar ratings to the items the users have rated in common. The set of users matched to the target user are referred to as the target user’s nearest neighbors. The method then recommends items to the target user that their neighboring users have rated highly. The assumption behind these methods is that if two users are observed to have close agreement on the ratings of a sufficiently large number of items, they will likely agree closely on the ratings for the remainder of the items.

**Figure 1:**
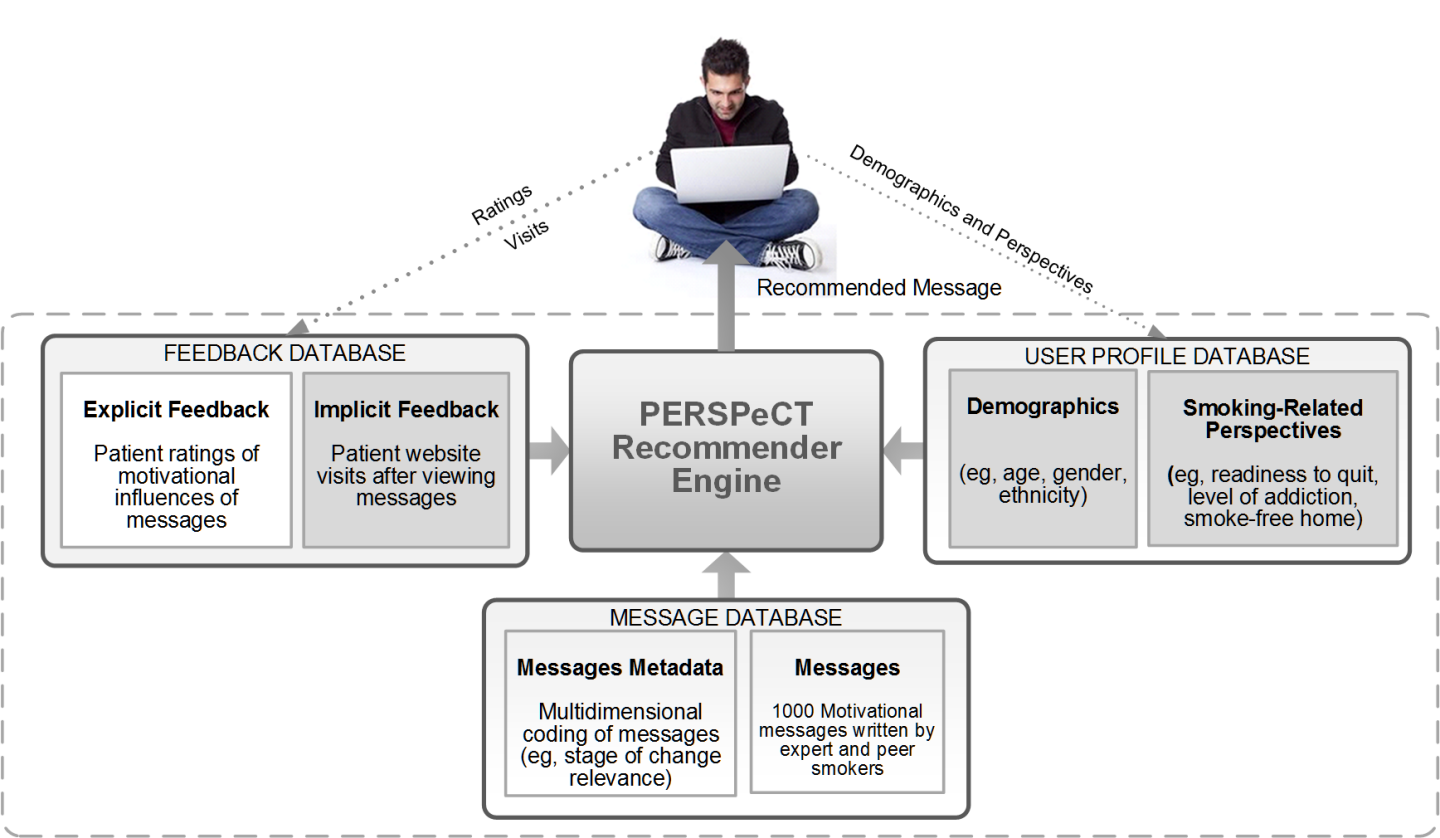
The Patient Experience Recommender System for Persuasive Communication Tailoring (PERSPeCT) recommender computer-tailored health communication system.

For PERSPeCT, we chose a hybrid approach because they merge the strengths of content-based and collaborative filtering recommender systems [22]. Thus, they can potentially benefit from expert-driven rules (content-based) and the recommender algorithms. We used the following data sources to develop the models for our algorithm: (1) metadata description of the messages, (2) implicit, and (3) explicit user feedback data (Figure 1). As explained previously, our coding of the messages by the readiness to quit categories is an example of metadata. In preparation for PERSPeCT, we expanded this metadata to include constructs from multiple behavioral theories, such as the SCT, the Transtheoretical Model, and the Theory of Reasoned Action [24]. We also coded the messages for content that may be pertinent to a specific user, including health and lifestyle status, health issues, and treatment options. In total, 40% (102/261) of messages had motivational content, such as reasons to quit, and 53% (139/261) of messages had information about behavioral treatments, such as substitution and distraction.

Implicit feedback data are derived from user actions (ie, website view patterns of each individual accessing the system). As our implicit feedback data, we used the website return data of 900 smokers that participated in our prior RCT [10]. When an email was sent to these smokers, we tracked their website usage in the days following the email. Thus, we had data on the frequency at which each message promoted engagement on the website and the characteristics of the smokers that received these messages.

Explicit feedback data consists of self-reported item ratings (ie, ratings provided by users for items like books or movies, often on a five-star scale). For companies such as Netflix, these are likely to be user ratings of movies. As previously published, two pilot studies were used to generate the explicit feedback data for PERSPeCT [16]. We first recruited 100 current or former smokers to determine appropriate questions for collecting explicit ratings. Each participant was asked to provide ratings using a five-point Likert scale of four different aspects of messages: influence, emotional response, relevance, and preference. Each participant provided ratings for five different randomly selected messages. Per-message analysis showed a positive correlation between the means and variances of the ratings for each question, suggesting that all questions provided similar information. Thus, we decided to use only one question for our data collection pilot, balancing the need to obtain multiple ratings per user and the resulting cognitive load. We chose the influence question stated in the data collection section because this single influence question had strong predictive validity in a previous RCT [25].

A second pilot test was performed to collect a larger rating dataset to bootstrap the learning and evaluation of collaborative filtering models for PERSPeCT [16]. We recruited 846 current or former smokers from online and local sources to provide perspectives on smoking, quitting, and sociocultural contextual information and ratings of the influential aspect of the 261 smoking cessation messages. Each smoker was asked to rate 20 messages, resulting in 16,920 ratings.

We tested a number of classical algorithms to identify one that provided maximal prediction accuracy (ie, we evaluated the ability of the algorithms to generalize ratings to nontraining users). These included the following algorithms: K-Nearest Neighbor (K-NN), probabilistic matrix factorization, Bayesian probabilistic matrix factorization (BPMF), collective matrix factorization, and Bayesian collective matrix factorization. We used a strong-generalization protocol that involved completely separating test users from train users, learning a model using all the train users’ ratings, freezing all nonuser-specific parameters, and finally training the user-specific parameters on a subset of each test user’s observed ratings. To implement this protocol, we first divided the users randomly into five folds and then generated three random train and validation sets for each test fold. We further divided each test user’s ratings into five folds. To evaluate each method’s performance given varying levels of information about a test user, we evaluated all methods with five, 10, and 16 of each test user’s ratings available for inference and learning of user-specific parameters. Each test user has a constant set of four test ratings per test fold. The validation sets were used to set the hyperparameters of each method (eg, K in K-NN). Exhaustive grid search was used and the hyperparameter ranges were iteratively extended to ensure that no selected hyperparameter values occurred at the end-points of the search intervals.

In evaluating rating prediction methods, we used a range of standard performance metrics including root mean squared error (RMSE), Kendall tau-b, and normalized discounted cumulative gain. In all these tests, BPMF was identified as the best single model in our evaluation and was used in the development of PERSPeCT. For example, comparing the RMSE metric between the different algorithms, there was a small but statistically significant gap (*P*=.01) between the BPMF and other algorithms as determined by a paired *t* test with Bonferroni correction. The BPMF model estimates a probability distribution over a joint embedding of users and items into complementary latent spaces. The rating a given user supplies for a given item is approximated by the expected value of the product of the latent user and item factor vectors representing the user-item pair, with the expectation taken over the uncertainty in embeddings. Since the algorithm that only included explicit ratings of the 846 smokers performed as well as the model that included all the data sources, for simplicity we chose to develop the model with only this explicit rating. The algorithm was also programmed to choose only from among those messages that matched the participant’s readiness to quit status. Further details regarding our algorithm selection methodology are described in previously published work [16].

### Setting and Sample

Current smokers were recruited from our University hospital and affiliated output clinics using multiple methods. We posted flyers at these clinics with instructions on how to contact the study staff. We worked with a tobacco treatment specialist to identify eligible smokers and refer them to the study staff. We also used electronic medical records to identify current smokers and mailed each smoker a letter describing the study and the contact information for the study coordinator. The letter explained that study staff would call them in 2 weeks to determine their interest and to answer any questions they had about the study. Included was a self-addressed, prestamped opt-out card that individuals could send back if they did not want to be contacted.

Current smokers who were 18 years of age or older, English speaking, and had Internet access were considered eligible for the study. To confirm participation, all smokers had to complete the online registration with the study staff over the phone. Smokers received a total of US $100 in Amazon gift cards for participation (US $25 for completing registration, US $25 for rating 15 messages, US $50 for completing the final survey and rating 30 messages).

### Randomization

**Figure 2:**
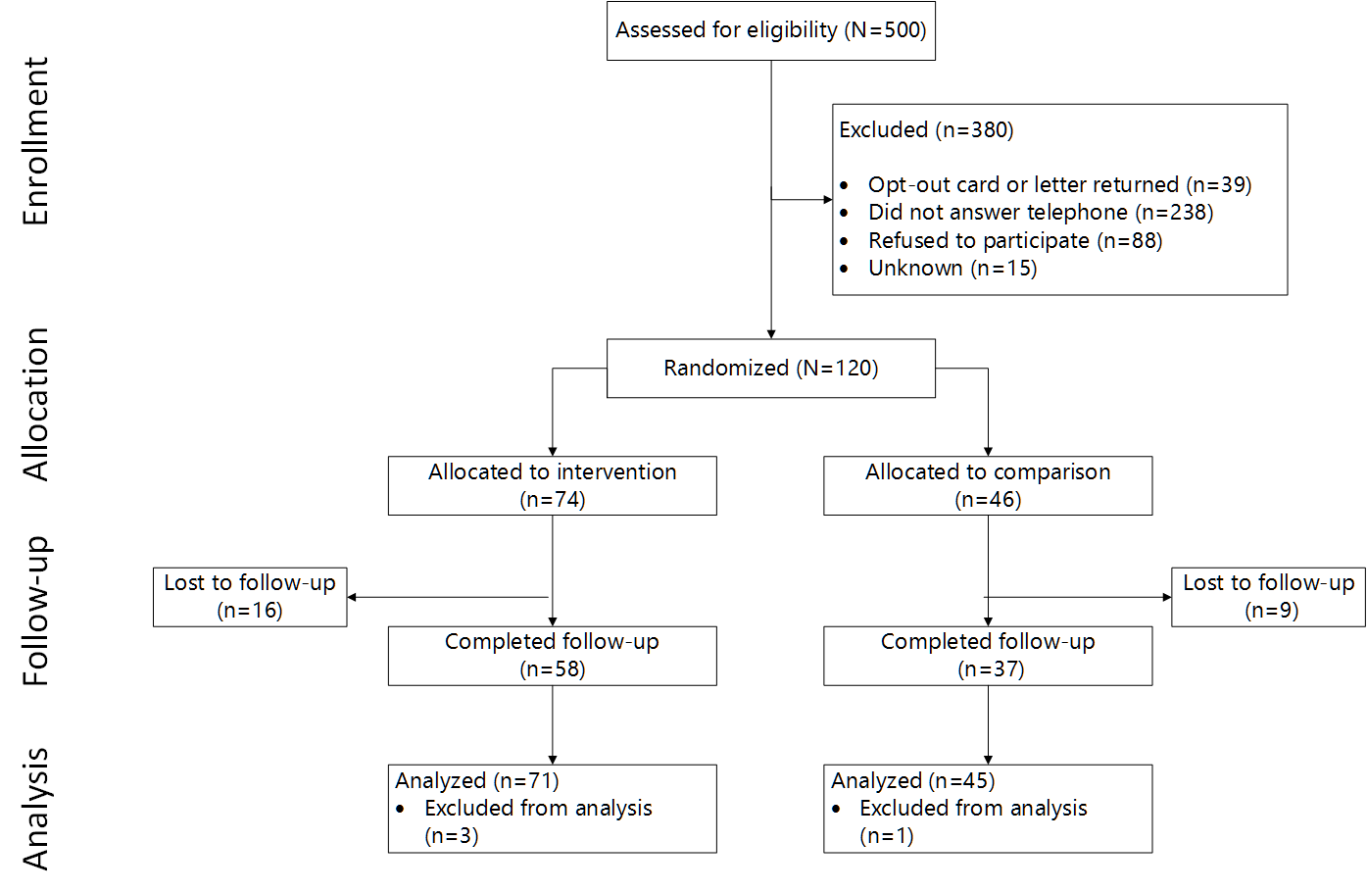
The Consolidated Standards of Reporting Trials (CONSORT) participant flow diagram.

As smokers registered online for our study, they were allocated to the two groups based on a prespecified, block-randomization allocation table (blocks of 10). Smokers were randomized to PERSPeCT or standard comparison in a 2:1 ratio (Figure 2). Unequal random allocation (favoring the intervention) increases experience with the experimental CTHC and can be desirable in early phase trials [26]. Because the standard system was proven effective and PERSPeCT was highly novel, 2:1 randomization allowed for additional subset analyses within the intervention group. Study staff was blinded to allocation during initial baseline assessment and follow-up.

### Data Collection

During registration, smokers were asked questions about their demographics (age, sex, race, and ethnicity), smoking behaviors, prior quit attempts, and readiness to quit [27,28]. Internet use was assessed using the following question: “For which of the following activities do you routinely use the Internet?” Message ratings were collected daily. Smokers were asked to rate each motivational email on a five-point Likert scale by clicking on a link included with the email. These ratings were collected for the standard system and PERSPeCT. For PERSPeCT, the system used the ratings to further improve message recommendations; for the standard system, the ratings were used only for analyses and did not to change the intervention. We used the following question to collect the rating: “This message influences me to QUIT smoking.”

At follow-up, the perceived influence of the intervention was assessed using seven questions adapted from prior measures of the influence of interventions on cessation [25]. We assessed 30-day cessation using the question: “Since starting the Quit Smoking Messaging System study have you stopped smoking for one day or longer because you were trying to quit?” Readiness to quit was assessed at baseline and the 30-day follow-up using the following options: I am not thinking of quitting, I am thinking of quitting, I have set a quit date, I quit today, and I have already quit.

### Statistical Analysis

All analyses were conducted using Stata version 13 (StataCorp LP, College Station, TX, USA). As noted, our primary hypothesis was that the PERSPeCT system would select messages of higher influence than a rule-based CTHC system. We also evaluated the perceived intervention influence, and 30-day cessation. For each analysis, we included all data available. For each individual, the timing of attrition varied. Note that if patients were lost to follow-up for the final 30-day outcome measurement, they would still have had data for daily ratings.

### Comparison of Message Ratings: Intervention Versus Control

For each day, we created a daily rating defined as the mean of the ratings provided by all smokers in the group that day. We then compared the daily ratings using a *t* test. To further explore the differences, we plotted a figure with the message day on the x-axis and the daily ratings on y-axis. We also compared the daily ratings stratified by the demographic characteristics.

#### Perceived Influence of the Intervention at 30 Days

We dichotomized the responses to each question that assessed the perceived influence of the intervention and used the chi-square statistic to test for differences. We conducted an additional sensitivity analysis of the perceived influence of the system stratified by the demographic characteristics (eg, age, gender, education, and readiness).

#### Cessation Influence

At 30 days, we evaluated change in smoking status compared to baseline. By randomization, we assessed change in smoking status (baseline to follow-up) using the chi-square statistic. Additionally, we assessed differences between the intervention and comparison groups of 30-day cessation using the chi-square statistic.

## Results

### Patient Characteristics

Smokers (N=120) were randomized to intervention (n=74) or comparison (n=46) (Figure 2). In total, 64.2% (77/120) of our sample were female, 38.3% (46/120) were aged 45 years or older, and 58.8% (70/120) were college graduate. There were no significant differences between the characteristics of intervention and comparison smokers (Table 1).

### Comparison of Message Ratings

We used all users with ratings for this analysis. Most users (77.5%, 93/120) rated all 30 messages. In answer to our primary hypothesis, the proportion of days when smokers agreed/strongly agreed (daily rating ≥4) that the messages influenced them to quit was significantly higher in the intervention (73%, 23/30) than comparison (44%, 14/30, *P*=.02).

**Figure 3:**
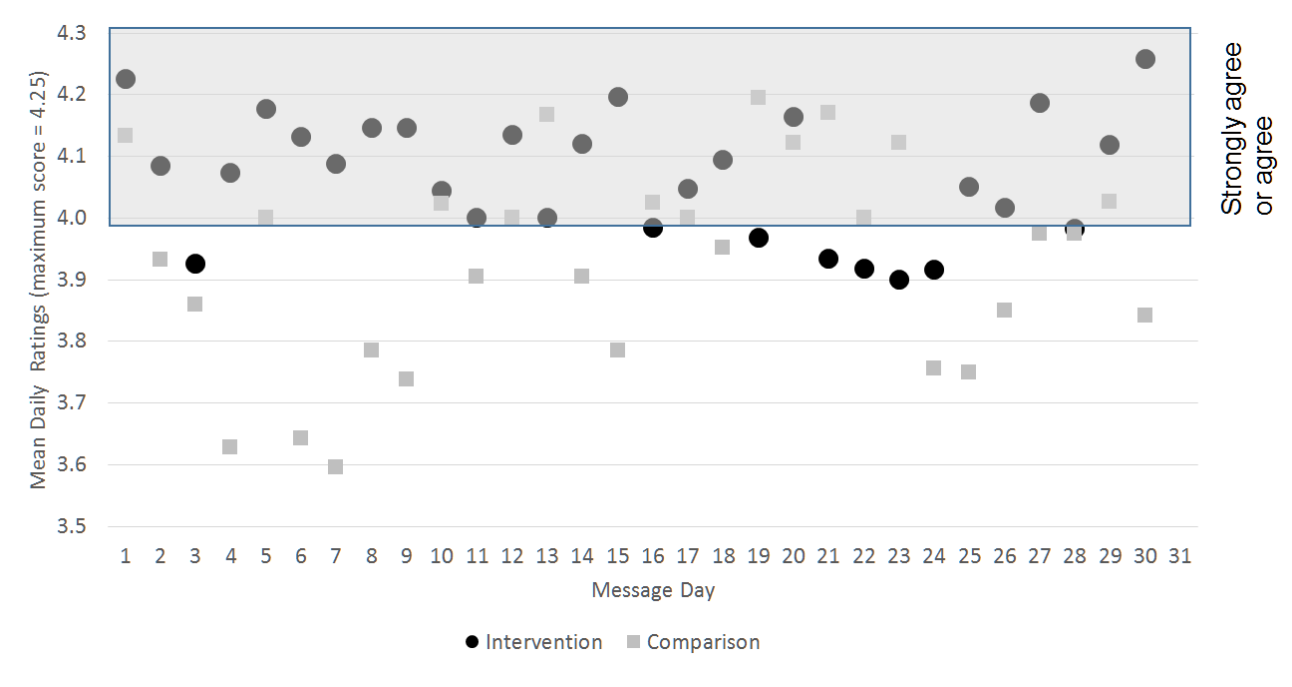
Mean daily ratings: intervention versus comparison.

Fluctuation of daily ratings of intervention smokers was less than that of the comparison group (Figure 3). Group differences of daily ratings were greatest within the first 12 days of the study (intervention: mean 4.10, SD 0.03; comparison: mean 3.86, SD 0.04; *P*<.001). Difference in the daily ratings between intervention and comparison declined over time (intervention: mean 4.05, SD 0.03; comparison: mean 3.98, SD 0.04; *P=*.12).

In our stratified analysis, we found that among less educated smokers (n=49), the difference in the proportion of days when smokers agreed/strongly agreed (daily rating ≥4) that the messages influenced them to quit was even more pronounced (intervention: 77%, 23/30; comparison: 23%, 7/30; *P*<.001).

**Table 1:** Demographic characteristics of participants.

Participant characteristics		Comparison, n (%) (n=46)	Intervention, n (%) (n=74)	Total, n (%) (N=120)	*P* value
**Sex**					.56
	Male	15 (33)	28 (38)	43 (35.8)	
	Female	31 (67)	46 (62)	77 (64.2)	
**Age (years)**					.45
	19-34	11 (24)	25 (34)	36 (30.0)	
	35-44	17 (37)	21 (28)	38 (31.7)	
	≥45	18 (39)	28 (38)	46 (38.3)	
**Education**					.83
	Less than high school	6 (13)	7 (10)	13 (10.9)	
	High school graduate	14 (30)	22 (30)	36 (30.3)	
	College graduate	26 (57)	44 (60)	70 (58.8)	
**Race**					.57
	White	43 (94)	67 (91)	110 (91.7)	
	Other	3 (7)	7 (9)	10 (8.3)	
**Hispanic or Latino**					.67
	No	36 (78)	62 (84)	98 (81.7)	
	Yes	4 (9)	6 (8)	10 (8.3)	
	Don’t know/not sure	6 (13)	6 (8)	12 (10.0)	
**Internet use (number of activities)**					.55
	No Internet use	2 (4)	2 (3)	4 (3.3)	
	0-2	12 (26)	12 (16)	24 (20.0)	
	2-4	6 (13)	12 (16)	18 (15.0)	
	>4	26 (57)	48 (65)	74 (61.7)	
**Allows smoking in home**					.62
	No	24 (52)	42 (57)	66 (55.0)	
	Yes	22 (48)	32 (43)	54 (45.0)	
**Visited a smoking cessation website**					.45
	No	36 (78)	62 (84)	98 (81.7)	
	Yes	10 (22)	12 (16)	22 (18.3)	
**Wants to stop smoking cigarettes**					.52
	No	9 (20)	13 (18)	22 (18.3)	
	Yes	37 (80)	59 (80)	96 (80.0)	
	I do not smoke now	0 (0)	2 (2)	2 (1.7)	
**Stopped smoking for one day or longer to try to quit smoking**					.68
	No	25 (54)	43 (58)	68 (56.7)	
	Yes	21 (46)	31 (42)	52 (13.3)	

### Perceived Influence of the Intervention at 30 Days

In total, 79.2% (95/120) of the smokers completed follow-up. Those lost to follow-up were equally distributed across both groups (intervention: 22%, 16/74; comparison: 20%, 9/46). There were no significant demographic differences between those that completed follow-up and those who did not. Among the users that completed follow-up, the perceived influence of the PERSPeCT system was higher than the comparison system in several categories, but not statistically significant. These include the perceived influence on the use of nicotine replacement therapy, such as the patch or gum (*P*=.09) and quit smoking (*P*=.07) (Table 2). In the sensitivity analyses, we did not find any significant or meaningful effect modification by demographic characteristics (recognizing that power was limited for this secondary exploratory analysis).

### Smoking Cessation at 30 Days

**Figure 4:**
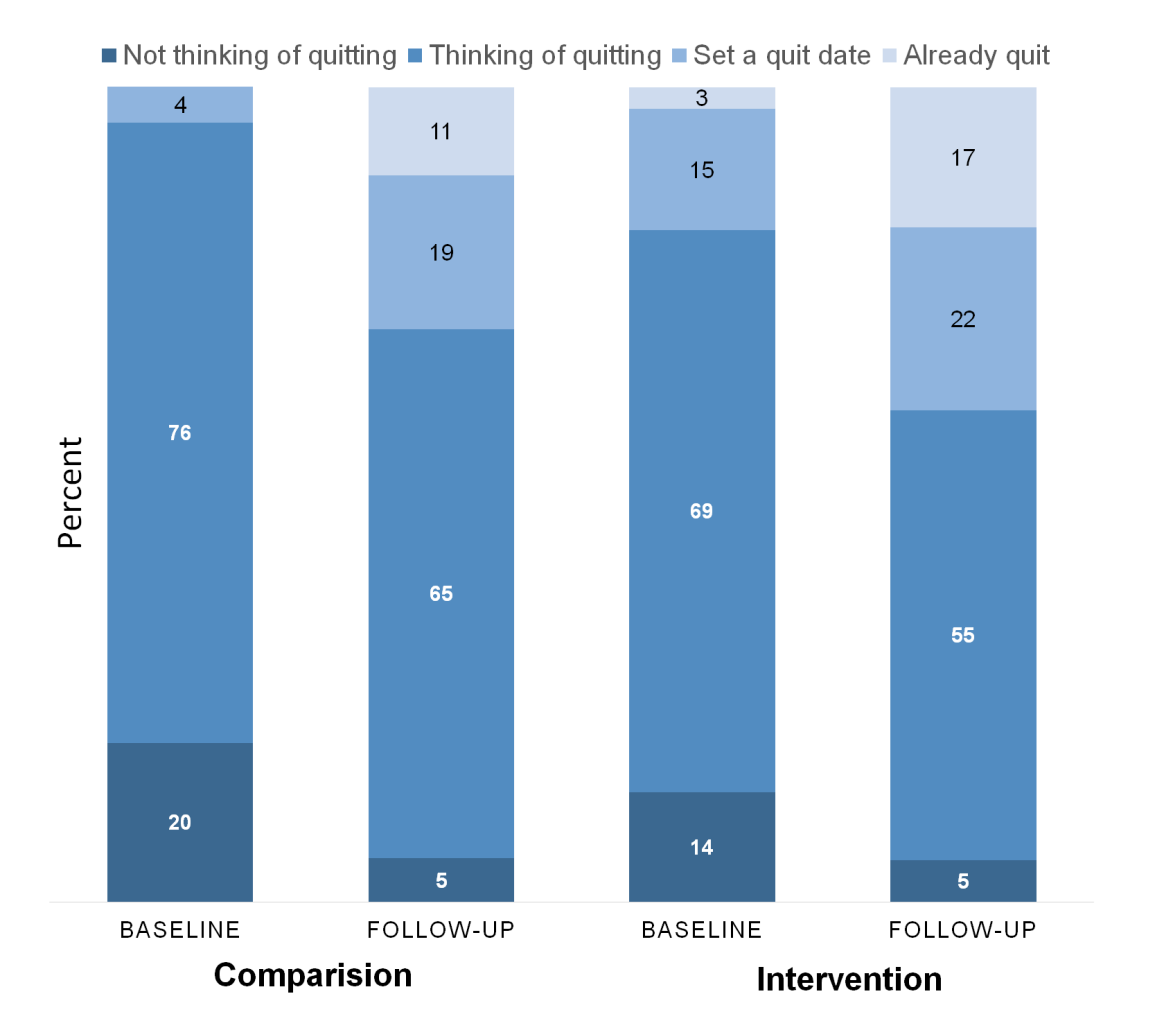
Baseline and follow-up readiness to quit status in percentages: PERSPeCT intervention (n=74) versus standard computer-tailored health communication comparison (n=46).

Among those who completed follow-up, 36% (20/55) of intervention smokers and 32% (11/34) of control smokers stopped smoking for one day or longer because they were trying to quit (*P*=.70). A higher proportion of intervention smokers reported that they had already quit or set a quit date (40%, 23/58 vs 30%, 11/37), but this did not meet statistical significance (Figure 4). In all, 35% (26/74) of participants in the intervention group and 30% (14/46) in the comparison group moved up the readiness-to-quit ladder (*P*=.60). The increase in the proportion of smokers who reported that they already quit in the intervention group was 15% and 11% in the comparison group.

**Table 2:** Influence of messages to participate in smoking cessation activities.

Perceived influence of the intervention		Comparison, n (%) (n=46)	Intervention, n (%) (n=74)	*P* value
**Use nicotine replacement therapy (eg, the patch or gum)**				.09
	Strongly disagree/disagree/neutral	20 (54)	21 (36)	
	Agree/strongly agree	17 (46)	37 (64)	
**Talk to a doctor about quitting smoking**				.39
	Strongly disagree/disagree/neutral	16 (43)	20 (34)	
	Agree/strongly agree	21 (57)	38 (65)	
**Quit smoking**				.07
	Strongly disagree/ disagree/neutral	14 (38)	12 (21)	
	Agree/strongly agree	23 (62)	46 (79)	
**Make a list of reasons to quit smoking**				.35
	Strongly disagree/disagree/neutral	10 (27)	11 (19)	
	Agree/strongly agree	27 (73)	47 (81)	
**Use behavioral strategies such as distraction or substitution**				.96
	Strongly disagree/disagree/neutral	7 (19)	11 (19)	
	Agree/strongly agree	30 (81)	47 (81)	
**Get support from those around you to help quit smoking**				.22
	Strongly disagree/disagree/neutral	9 (24)	21 (36)	
	Agree/strongly agree	28 (76)	37 (64)	
**Set a quit date**				.42
	Strongly disagree/disagree/neutral	23 (62)	28 (48)	
	Agree/strongly agree	14 (38)	30 (52)	

## Discussion

We developed a novel machine learning recommender system (PERSPeCT) directly driven by user feedback. In a small randomized experiment using the same database of motivational messages, the message selections produced by the new recommender system outperformed a robust rule-based standard CTHC system (previously demonstrated to be effective) in terms of both daily mean rating and self-reported intervention influence. At 30-day follow-up, a higher proportion of intervention smokers also reported a change in status to already quit or set a quit date, and 30-day cessation.

The ultimate goal of our CTHC intervention was to increase motivation and influence cessation. We tested this in a number of ways: (1) daily ratings of messages (hypothesis), (2) perceived influence of the intervention, and (3) cessation behavior. Comparing the daily ratings of the two systems, messages selected by the PERSPeCT system had more statistically significant days with mean ratings higher than 4 (agree or strongly agree) than the comparison system. In particular, during the initial messaging days, the daily ratings of the PERSPeCT messages were consistently higher than the ratings of the comparison system. As prior studies have demonstrated, most technology interventions suffer from high attrition rates, with the use highest in the initial days [29-34]. The ability to engage and motivate participants in the initial days is crucial to the success of an intervention. The potential ability of PERSPeCT to select messages of higher influence during the initial messaging period might be an important advantage over a standard CTHC system and this needs to be further tested.

Even in the short time span of our study (30 days) and compared with an effective rule-based CTHC, the PERSPeCT system demonstrated a greater influence on cessation behavior. Although not significant, more users in the intervention reported that they had a positive change in readiness to quit. More smokers in the intervention also reported that they had stopped smoking for one day or longer because they were trying to quit. A larger RCT is needed to test these promising results further.

Our study has some limitations. The goal of the study was to demonstrate feasibility and potential of PERSPeCT (comparing the system to a known effective system). Our comparison system has demonstrated effectiveness on long-term smoking outcomes, but we did not assess 6-month point prevalence cessation in this study, assessed only short-term quit outcomes. Thus, we are limited to surrogate outcomes (ratings of influence) that have been demonstrated in prior work to be associated with longer-term cessation. Our smaller sample size was driven by our primary hypothesis (differences in ratings). In this study, we only compared to one other system. Although this enhanced the internal comparison and isolated the tailoring algorithm effect, our results may not be generalizable to other systems. Before conducting a definitive trial of outcomes for a novel technology with lack of prior research, it is appropriate to conduct a smaller experiment to demonstrate effect on more proximal outcomes, justifying the larger trial. Further, our patients may not be representative of all smokers. Note that we delivered our messages only in English. In addition, many smokers who do not have Internet access would not be able to receive the motivational emails. These smokers would benefit from translation of the system into another commonly available communication format, such as texting.

In conclusion, recommender systems have not been applied to CTHC and our paper demonstrates that recommender systems can improve performance of CTHC. There are several reasons for this improved performance [11]. A primary reason is that recommender systems can learn and adapt to a participant’s behavior, whereas standard CTHC adapt only to predicted changes in behavior (ie, based on identified tailoring variables and rules). In our experiment, PERSPeCT adapted to the daily ratings (ie, explicit feedback) of the smoker. Future versions can be also be developed to adapt to the implicit behavior of a smoker receiving the messages. Leaders in the field of CTHC have demonstrated that high tailoring (tailoring on many variables) is better than low tailoring (using fewer variables) [8]. Rule-based standard CTHC systems have limitation in the number of variables that can be incorporated [11], whereas sophisticated machine learning algorithms may be able to tailor use of all available user variables and tailor based on these variables. Recommender systems also augment theory-based approaches because they would identify important variables from user data and behavior. Our small experiment successfully demonstrates the potential of the PERSPeCT system and highlights the need for larger trials to assess its true impact.

## Appendix

Multimedia Appendix 1 CONSORT 2010 checklist of information.

## Abbreviations

BPMF: Bayesian probabilistic matrix factorization
CTHC: computer-tailored health communication
K-NN: K-Nearest Neighbor
PCORI: Patient-Centered Outcomes Research Institute
PERSPeCT: Patient Experience Recommender System for Persuasive Communication Tailoring
RCT: randomized controlled trial
RMSE: root mean squared error
SCT: Social Cognitive Theory
